# Supplementary material for: Hydrogel-embedded vertically aligned metal-organic framework nanosheet membrane for efficient water harvesting
Source: Nat Commun. 2024 Nov 11;15:9738. doi: 10.1038/s41467-024-54215-z (PMC11555079; doi:10.1038/s41467-024-54215-z)
Supplement: Supplementary file 1 — Supplementary Information [file 41467_2024_54215_MOESM1_ESM.pdf]

SUPPLEMENTARY INFORMATION

Hydrogel-embedded Vertically Aligned Metal-Organic Framework Nanosheet Membrane for Efficient Water Harvesting

Lingyue Zhang[1], Ruiying Li[1], Shuang Zheng[1], Hai Zhu[1], Moyuan Cao[2], Mingchun Li[3], Yaowen Hu[1], Li Long[1], Haopeng Feng[1], Chuyang Y. Tang[1, *]

1 Department of Civil Engineering, The University of Hong Kong, Pokfulam, Hong Kong SAR 999077, China

2 School of Materials Science and Engineering, Smart Sensing Interdisciplinary Science Center, Nankai University, Tianjin, 300350 P.R. China

3 School of Environment, Tsinghua University, Beijing 100084, China

The supplementary information consists of 33 pages, 21 figures and 5 tables.

Supplementary Methods

Materials

Zinc nitrate hexahydrate ($Zn(NO_3)_2 \cdot 6H_2O$, 98%), poly(ethylene glycol) (PEG, average molecule weight 20,000), N,N-Dimethylformamide (DMF, 99.8%), hydrochloric acid (HCl, 36-38%), sodium hydroxide (NaOH, > 98%) were purchased from Sigma-Aldrich. Tetrakis(4 carboxyphenyl)porphyrin (TCPP, 97%) and poly(vinyl alcohol) (PVA, n = approx. 1700) was purchased from Tokyo Chemical Industry Co. Ltd. Chitosan (CT, molecule weight 30,000) was purchased from Macklin. Ethanol (> 99.9%) was purchased from VWR Chemicals. Pyrazine (99%) was purchased from Alfa Aesar. Diiodomethane (CH_2I_2, 98%) was purchased from Aladdin. The deionized water was obtained from the Milli-Q System. All the materials were used as received without further purification.

Synthesis of bulk Zn-TCPP

The compared bulk Zn-TCPP was fabricated based on literature [1]. 8.9 mg $Zn(NO_3)_2 \cdot 6H_2O$, 7.9 mg TCPP, and 1.6 mg pyrazine were dissolved in the mixture of DMF (1.5 mL) and ethanol (0.5 mL) and heated at 80 °C for 24 h. The resulting purple bulk crystals were washed with ethanol and collected by centrifuging at 7,100 g for 10 min.

Preparation of detached nanosheet Zn-TCPP

The nanosheet Zn-TCPP used in this study was detached from the MOF-CT/PVA. The purple nanosheets on the surface of the MOF-CT/PVA were scraped off using tweezers and sonicated for 10 minutes to disperse them in ethanol. The resulting nanosheets were washed thrice with ethanol and collected by centrifugation at 7,100 g for 10 minutes.

Preparation of control PEG@CT/PVA membranes

We fabricated an additional membrane, PEG@CT/PVA, to study the role of PEG in water adsorption. Specifically, 72 mg of PEG was dissolved in a mixture of DMF and

ethanol (V:V=3:1) to a total volume of 48 mL, mirroring the PEG amount used in the MOF-CT/PVA. The CT/PVA hydrogel membrane substrate was then immersed in this solution at 80 °C and subjected to continuous agitation for 24 hours. The resulting membrane was rinsed three times with ethanol, and this membrane is referred to as PEG@CT/PVA. The water adsorption results are presented in Supplementary Fig. 21.

Preparation of horizontally stacked MOF membranes

By calculating the mass difference of the MOF-CT/PVA membrane before and after MOF growth, detached Zn-TCPP nanosheets of the same quality were filtered onto the CT/PVA substrate to prepare a horizontally stacked MOF membrane. The obtained membrane harvester is abbreviated as HMOF-CT/PVA.

Calculation of the surface free energy of membranes

The surface free energy of hydrogel membranes was determined using the Owens, Wendt, Rabel, and Kaelble method, employing the contact angles of water and diiodomethane. In accordance with Young's equation, the surface free energy σ_s of the solid was calculated using the following equation:

$$\sigma_s = \sigma_{sl} + \sigma_l \cdot \cos\theta \tag{1}$$

where, θ is the contact angle, σ_l is the surface tension of the liquid, σ_{sl} is the interfacial tension between liquid and solid.

The interfacial tension σ_{sl} is calculated based on the Fowkes method, taking into consideration the two surface tensions σ_s and σ_l and the similar interactions between the phases.

$$\sigma_{sl} = \sigma_s + \sigma_l - 2\left(\sqrt{\sigma_s^D \cdot \sigma_l^D} + \sqrt{\sigma_s^P \cdot \sigma_l^P}\right) \tag{2}$$

where, σ^D is the geometric mean of a disperse part, σ^P is a polar of the surface tension.

Simulation methods of water transportation between MOF nanosheets

A two-phase flow simulation method was employed to investigate the movement of two immiscible fluids, specifically focusing on accurately determining the interface between water and air. As an integral component of computational fluid dynamics (CFD), this method takes into account the variations in density and viscosity between the two fluids, while also considering the impact of surface tension. To simulate the transportation of water between MOF nanosheets, a simplified two-dimensional flow field was used, representing the vertically aligned and horizontally stacked MOF layer. The contact angle between water and the structural surface was set at 5°. The upper portion of the model was designated as liquid, while the structure was surrounded by air. The liquid was transported from top to bottom. The volume fraction of air was set at 1 (indicated by the red area in Fig. 3b), the volume fraction of liquid was 0 (indicated by the blue area in Fig. 3b), and the volume fraction of the gas-liquid interface was 0.5 (indicated by the yellow area in Fig. 3b).

The process of air-liquid interface evolution was calculated by the following equations.

$$\rho\frac{\partial u}{\partial t} + \rho(u\,\nabla)u = \nabla\,[-pI + \mu(\nabla u + \nabla u^{\mathrm{T}})] + F_{\mathrm{st}} + F \quad (3)$$

$$\nabla\, u = 0 \quad (4)$$

where, ρ is the fluid density, u is the fluid velocity, F_{st} indicates the surface tension acting at the interface between the two fluids, F is the volume or body force which acts through the volume of the body.

$$F_{\mathrm{st}} = \sigma\delta\kappa n + \delta\nabla_{\mathrm{s}}\sigma \quad (5)$$

where, σ is the surface tension coefficient (N m^{-1}), δ (m^{-1}) is a Dirac delta function on the interface and $\kappa = -\nabla\, n$ is the curvature, n is the unit normal to the interface, ∇_s is the surface gradient operator.

Dynamics simulation methods of water droplets transportation between MOF nanosheets

To further understand the dynamics of compensated droplets within the channels formed by varying vertical distributions of nanosheets, an iterative method was

employed in a simplified two-dimensional model. Based on the Young-Laplace equation, the capillary pressure exerted on water droplets was found to be inversely proportional to the diameter of the pore channels. Consequently, a simplified numerical integration of equations (6)-(8) was performed to calculate the localized profiles of velocities *v*, accelerated velocities a, and normal transport distances L by using solve function in Matlab software (R2021b, Mathworks, United States, Sep 2021). By using iteration method, the variables within this segment were continually updated and utilized as feed inputs for subsequent segments.

$$a_i = k/(d_f + \frac{L_{i-1}}{L_t} \cdot (d_p - d_f)) \tag{6}$$

$$v_i - v_{i-1} = a_{i-1} \cdot t \tag{7}$$

$$L_i - L_{i-1} = v_{i-1} \cdot t \tag{8}$$

where, d_f is the entry length, d_p is the outlet length, L_t is the total transport distance in the normal direction, k is the surface tension coefficient, and *t* is the simulation time.

Supplementary Figures

a

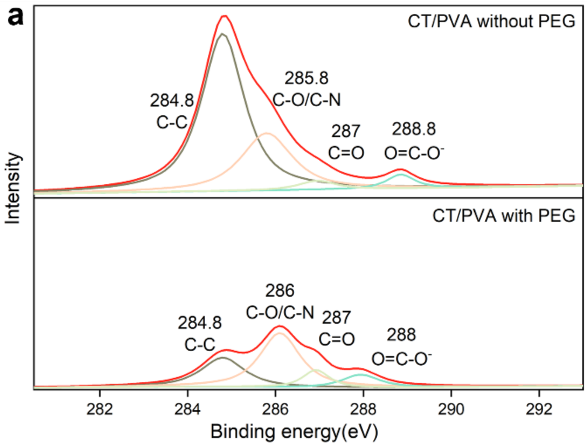

b

Materials	C 1*s*			
	C-C	C-O/C-N	C=O	O=C-O
CT/PVA without PEG	41.3	27.5	15.5	15.7
CT/PVA with PEG	25.5	41.9	16.3	16.2

Supplementary Fig. 1. a, XPS C 1*s* spectrum and **b,** relative content (%) of CT/PVA hydrogel membranes before and after crosslinking reaction with PEG.

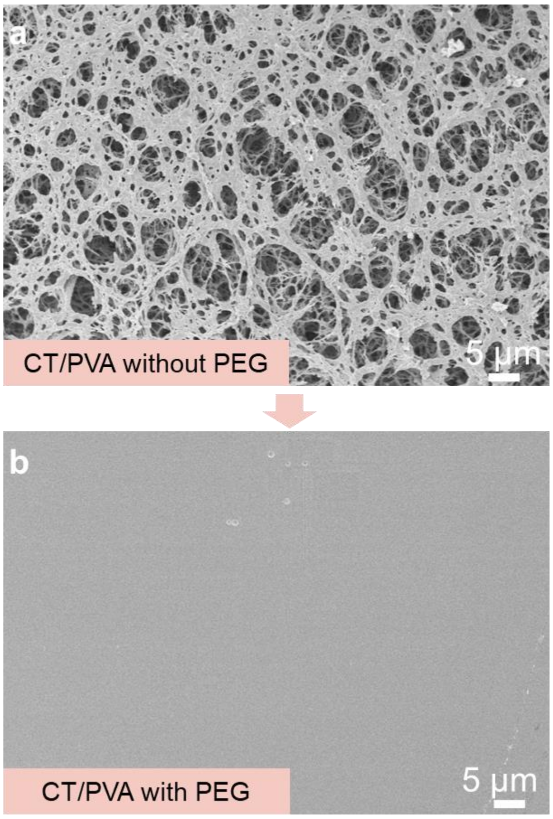

Supplementary Fig. 2. SEM images of the surface of CT/PVA hydrogel membranes **a,** before and **b,** after crosslinking reaction with PEG under MOF solvent system.

Prior to SEM characterization, the CT/PVA membrane substrate both before and after crosslinking with PEG underwent a 24-hour immersion in a solvent system comprising DMF and ethanol (V:V=3:1). Surface images (Supplementary Fig. 2) revealed that the CT/PVA hydrogel membrane was extensively dissolved in the MOF solvent system, exposing the bottom layer of non-woven fabric on the membrane surface. In contrast, limited changes and no significant dissolution of the hydrogel substrate were observed in the morphology of CT/PVA with PEG. These findings suggest that the presence of PEG and the crosslinking reaction are crucial factors in maintaining the stability and integrity of the hydrogel substrate during the directional growth of Zn-TCPP.

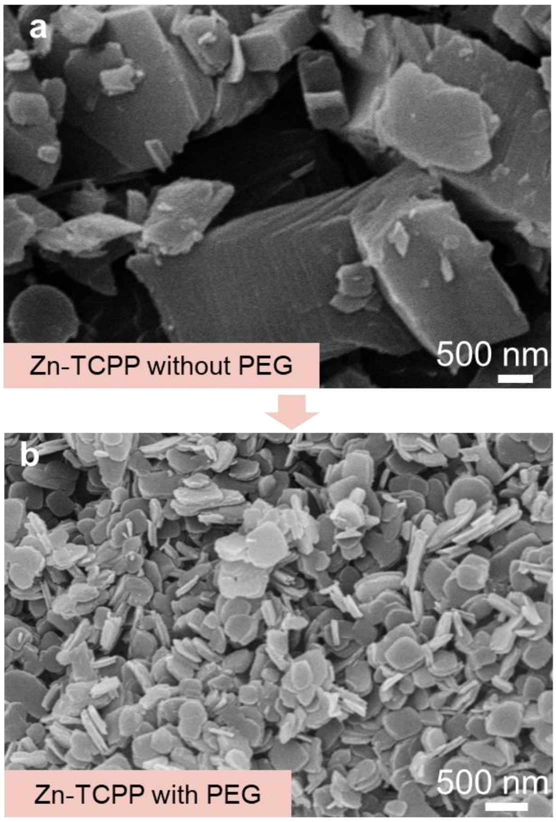

Supplementary Fig. 3. Nanoparticle SEM images of **a,** bulk Zn-TCPP and **b,** Zn-TCPP with the presentation of PEG.

Without PEG as non-ionic surfactants to control the anisotropic growth process, a bulk crystal of Zn-TCPP was synthesized, as depicted in Supplementary Fig. 3. The presentation of PEG results in disc-like nanoparticles with a multilayer thickness of approximately 100 nm, indicating the integrated anisotropic growth of Zn-TCPP nanosheets with the PEG adsorption. However, this disc-like morphology differed significantly from the petal-like morphology, which was only 10 nm in thickness and observed on MOF-CT/PVA. The presence of PEG in a directed arrangement at the interface of hydrogel membrane further influences the rectangular shape of Zn-TCPP.

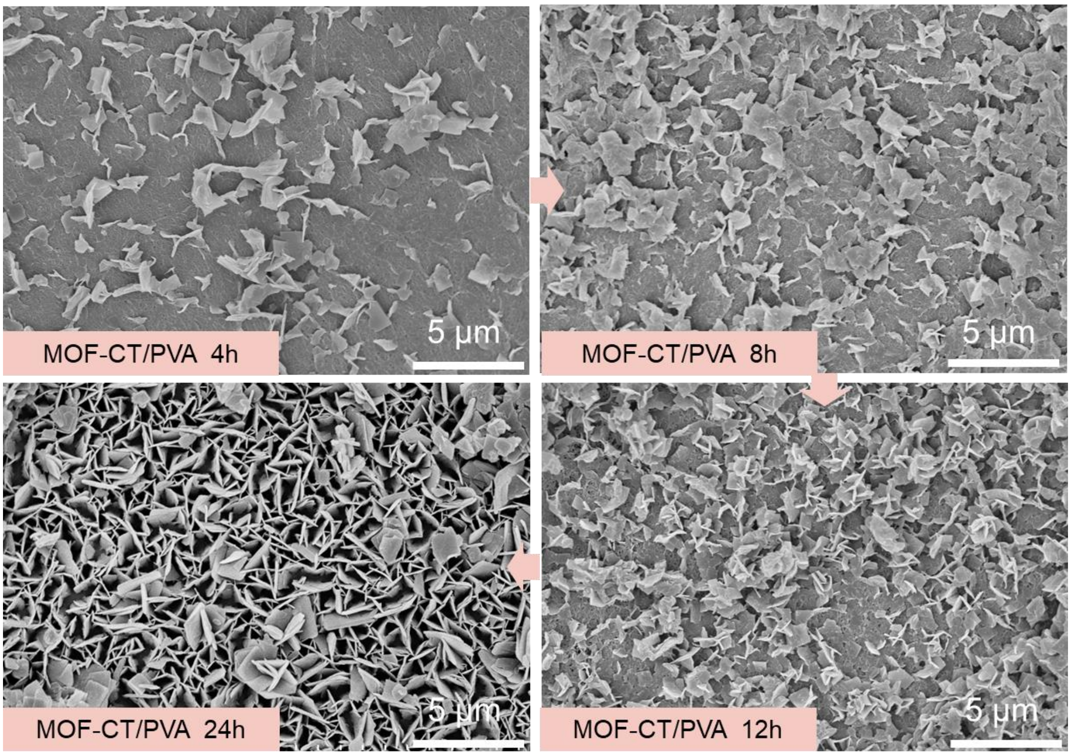

Supplementary Fig. 4. Surface morphologies of MOF-CT/PVA during the growth process at 4h, 8h, 12h, and 24h.

In contrast to previously reported stacking or bulk-shaped Zn-TCPP nanosheets, a continuous and uniformly vertically aligned nanosheet layer was formed during the membrane formation process. This growth process further demonstrated that the morphology of the CT/PVA membrane substrate remained constant throughout the synthesis process of the MOF layer. The in-plane orientation growth observed within the first 8 hours indicated the occurrence of the Van der Drift competitive growth theory in this direction, resulting in the growth orientation gradually changing to the low-resistance vertical direction.

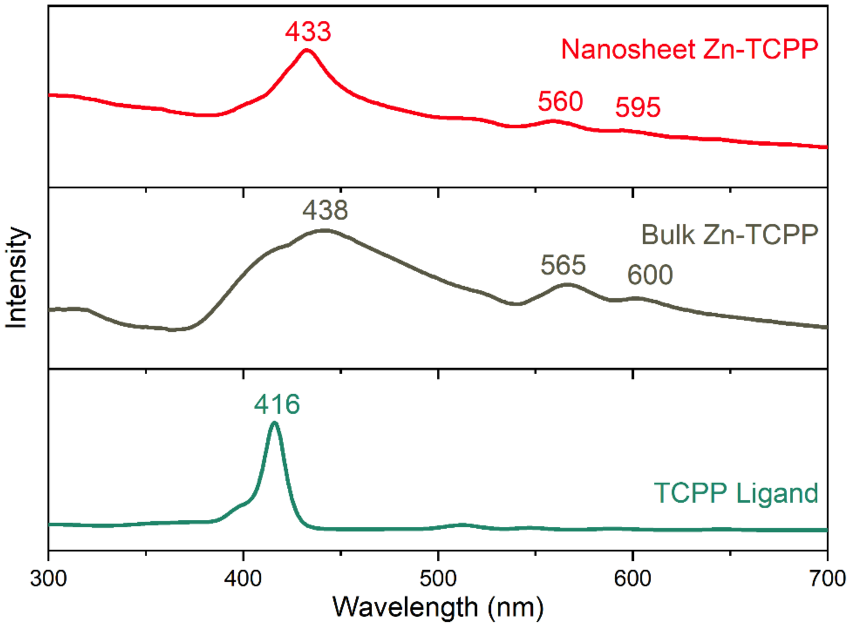

Supplementary Fig. 5. UV-vis absorption spectra of nanosheet Zn-TCPP, bulk Zn-TCPP, and TCPP ligand in ethanol.

The characteristic peak of porphyrin in the free TCPP ligand at 416 nm red shifted to 433 nm in the Zn-TCPP nanosheets with two predominant Q bands at 560 nm and 595 nm, which align with those observed in bulk Zn-TCPP.

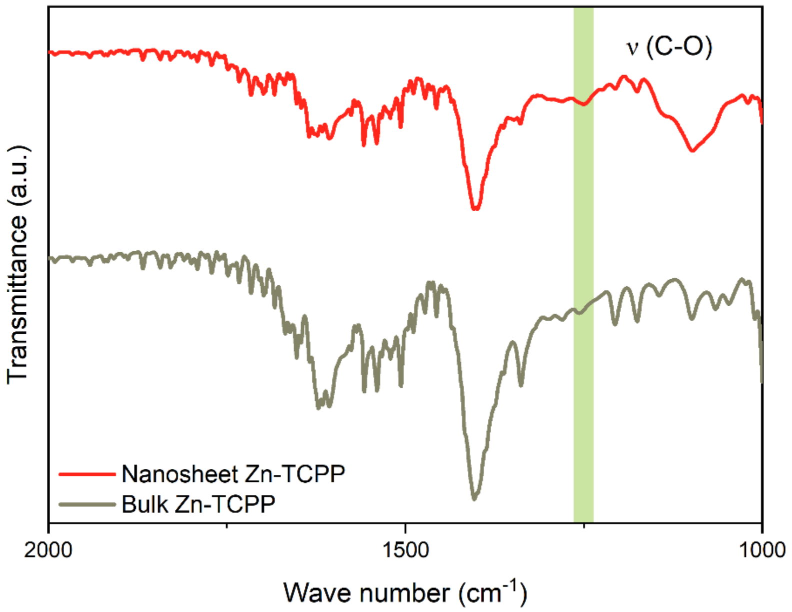

Supplementary Fig. 6. FTIR spectra of nanosheet Zn-TCPP and bulk Zn-TCPP.

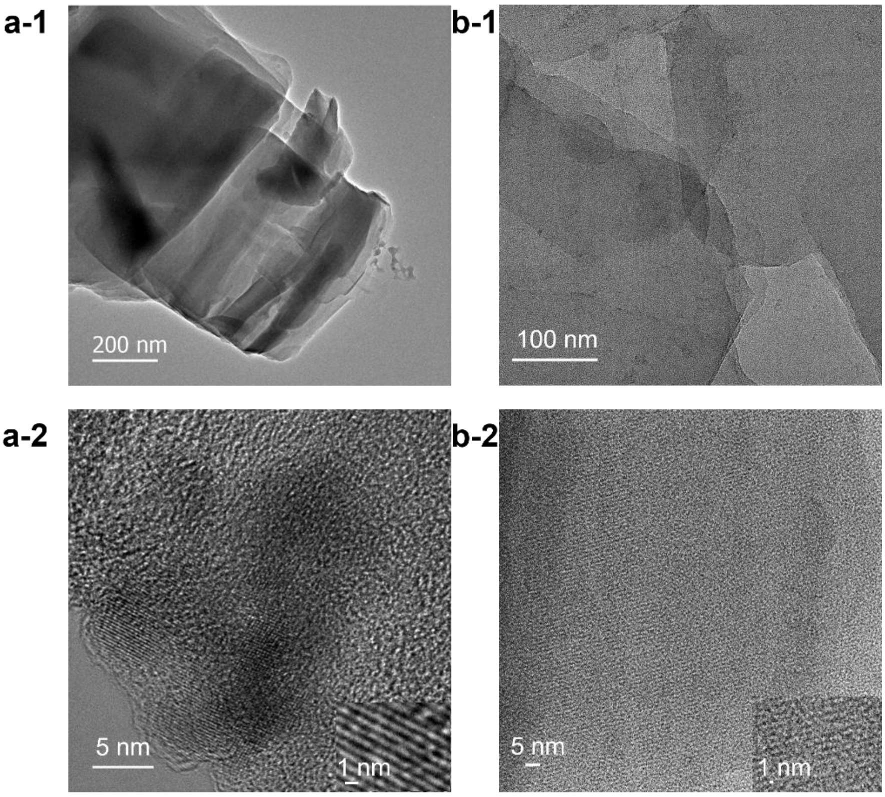

Supplementary Fig. 7. HR-TEM images of **a-1,** bulk Zn-TCPP and **b-1,** nanosheet Zn-TCPP. HR-TEM image of **a-2**, a single bulk Zn-TCPP and **b-2,** a single nanosheet Zn-TCPP. Inset: HR-TEM images for the clear crystal lattice.

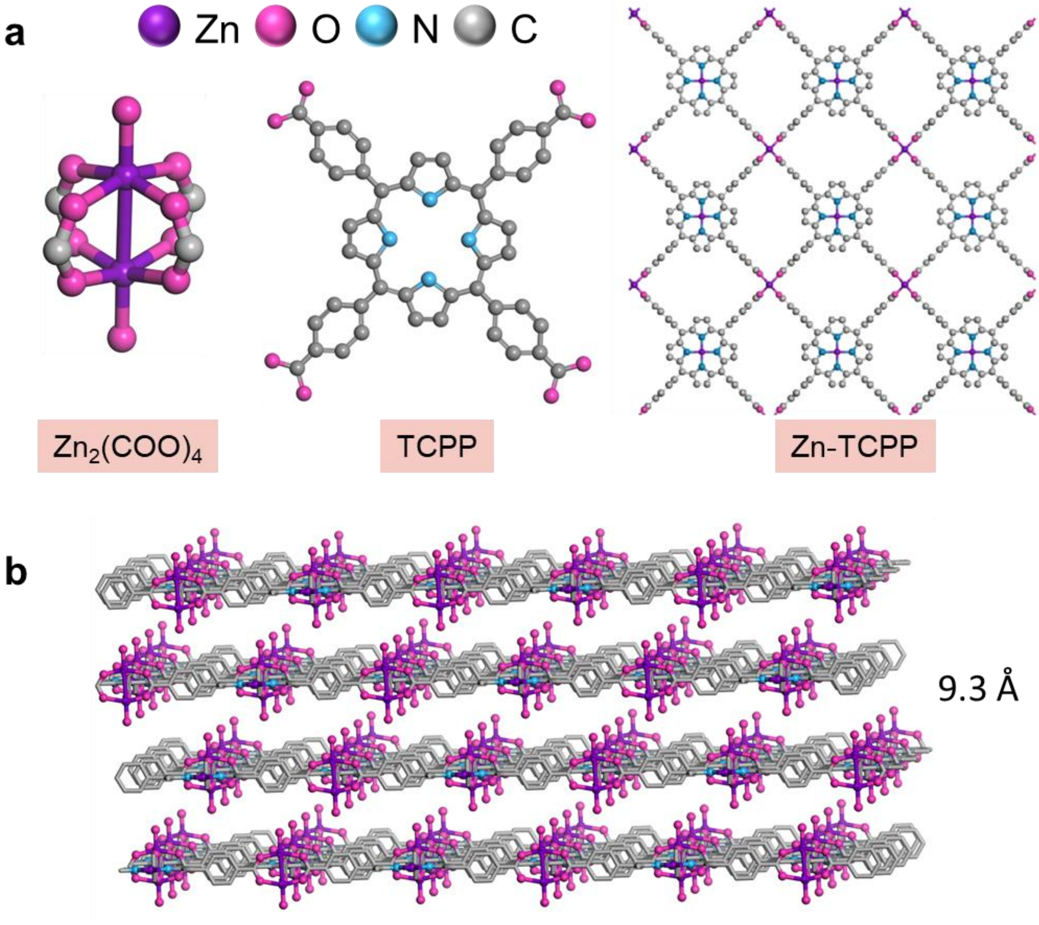

Supplementary Fig. 8. Crystal structure of Zn-TCPP nanosheets. **a**, Structures of $Zn_2(COO)_4$ paddlewheel metal node, TCPP ligand, and the constructed Zn-TCPP nanosheet. **b**, The layered structure of Zn-TCPP nanosheets with an interlayer distance of 9.3 Å at 296 K [1].

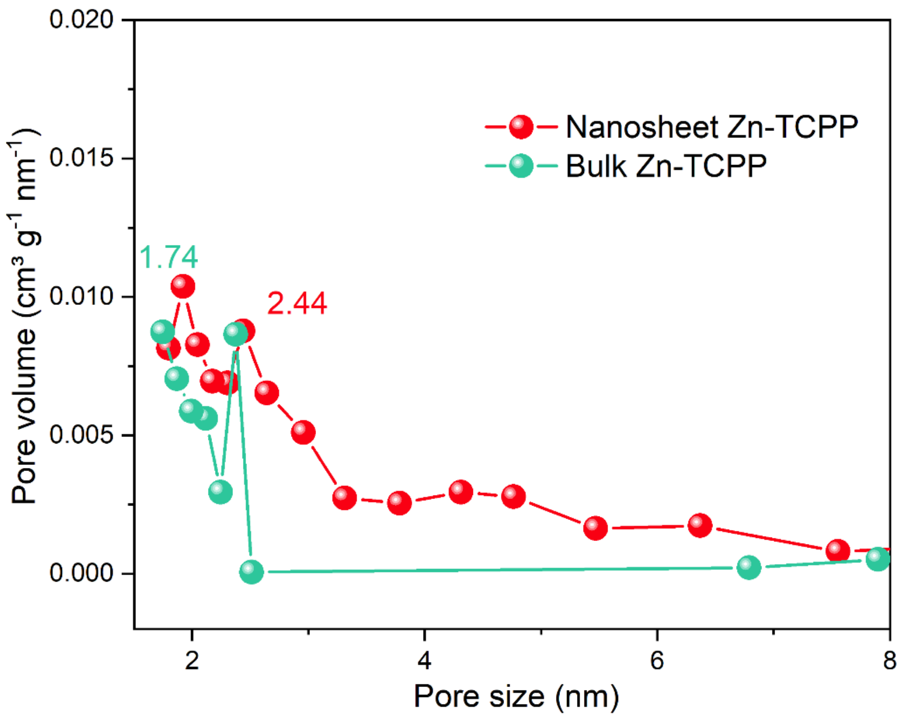

Supplementary Fig. 9. Pore size distribution curves of bulk Zn-TCPP and nanosheet Zn-TCPP. The BJH equations were used to calculate the pore size destitution.

The pore size distribution data obtained revealed that both the Zn-TCPP nanosheets and bulk Zn-TCPP exhibited similar micropores with a diameter of approximately 1.74 nm. This finding is consistent with the value of 1.67 nm determined from crystallographic data [2]. However, the Zn-TCPP nanosheets also exhibited additional pores in the range of 2.44 - 3 nm. These larger pores could be attributed to the formation of slit-like pores resulting from the aggregation of Zn-TCPP nanosheets during the freeze-drying process prior to the BET characterization. The freeze-drying process can induce the aggregation of nanosheets, leading to the formation of interconnected slit-like pores between the nanosheet layers.

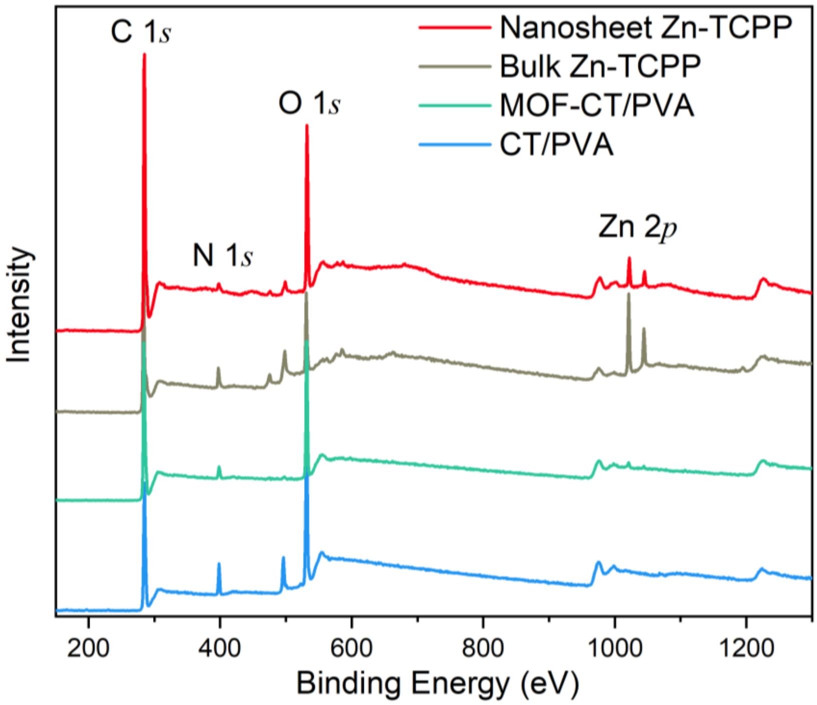

Supplementary Fig. 10. The XPS spectra of nanosheet Zn-TCPP, bulk Zn-TCPP, MOF-CT/PVA, and CT/PVA substrate. The Zn 2*p* characteristic peaks were observed in the XPS spectrum of nanosheet Zn-TCPP, bulk Zn-TCPP, and MOF-CT/PVA.

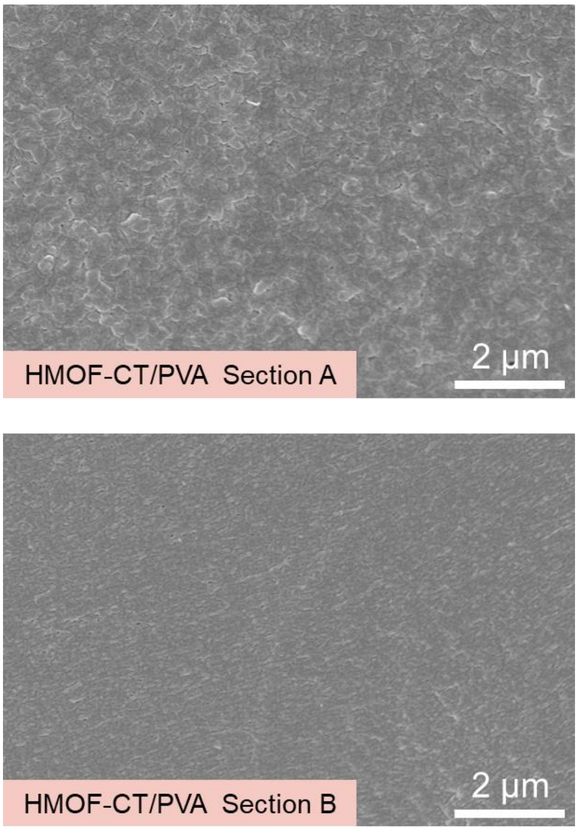

Supplementary Fig. 11. SEM images of the different surface sections of HMOF-CT/PVA hydrogel membrane.

It is important to note that despite the ultrasonic dispersion of the solution system of HMOF-CT/PVA beforehand, agglomeration and stacking phenomena unavoidably occur during the nanosheet filtration process, making it nearly impossible to form a continuous nanosheet layer on the substrate membrane under identical weight conditions. This phenomenon has been frequently reported in the literature, even with a significant number of nanoparticles [3, 4].

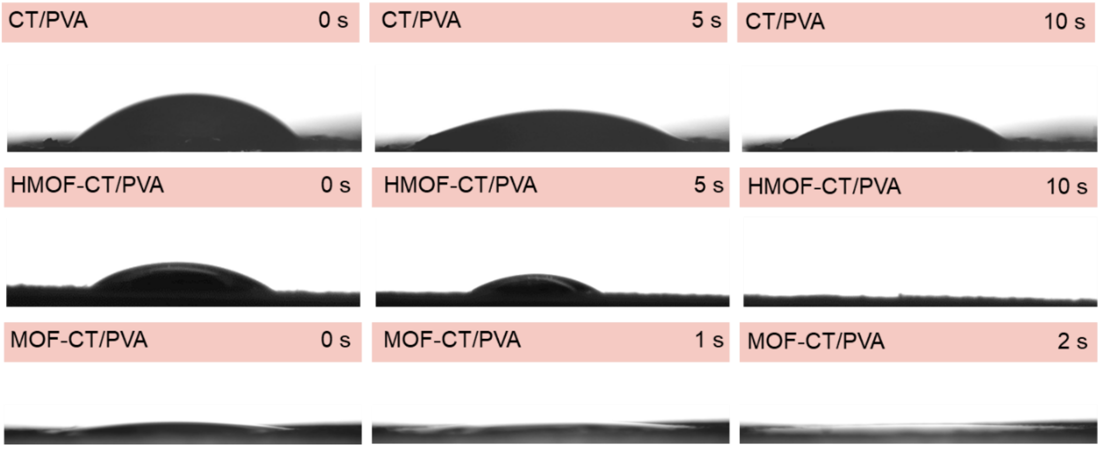

Supplementary Fig. 12. Changes in water contact angle of CT/PVA, HMOF-CT/PVA, and MOF-CT/PVA over time.

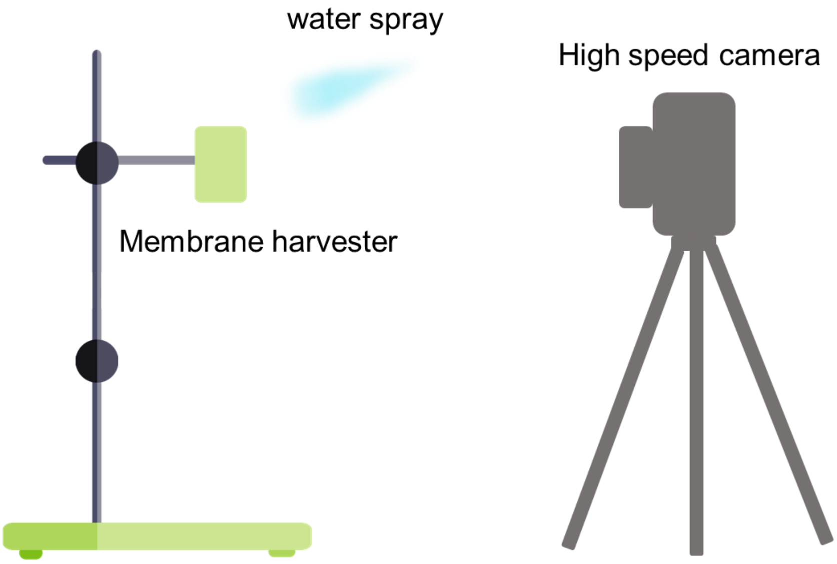

Supplementary Fig. 13. Schematic of the wetting process measurement of sprayed water droplets using high-speed camera.

All test membranes were positioned at a specific location in alignment with the high-speed camera. A consistent amount of 0.5 mL of water was sprayed each time, with the spraying source fixed at a distance of 30 centimeters from the membranes throughout the experiment.

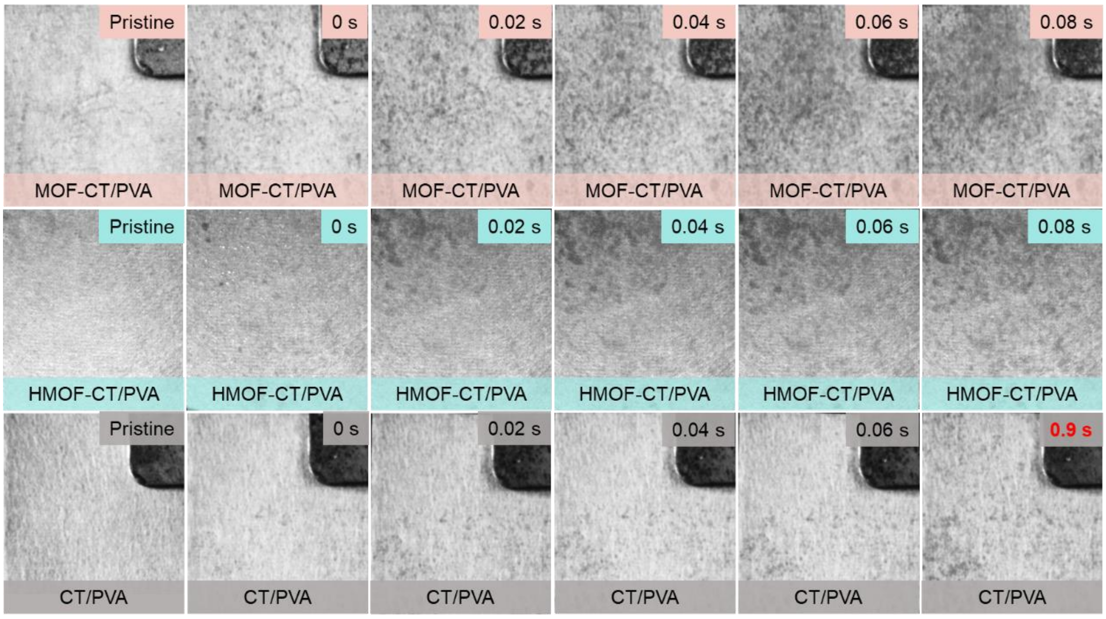

Supplementary Fig. 14. Optical images of the droplet capture, coalescence, and directional transportation process of the hydrogel membranes taken by high-speed camera. Inset labels: time intervals after water droplets were sprayed onto the membrane samples.

Upon initial water mist spraying onto the membranes, MOF-CT/PVA exhibited rapid absorption of small water droplets, resulting in distinct dark black wetting dots. In contrast, HMOF-CT/PVA and CT/PVA surfaces showed unabsorbed transparent water droplets. As the adsorption time increased, the water droplets coalesced, diffused, and gradually became absorbed into the hydrogel, forming a wetting surface. This absorption process occurred at an ultrafast rate on the hydrogel membrane with the MOF layer, particularly on MOF-CT/PVA, but was significantly slower on the CT/PVA substrate. Even after 0.9 seconds, only a small portion of the CT/PVA surface was wetted and gradually tended to dry out.

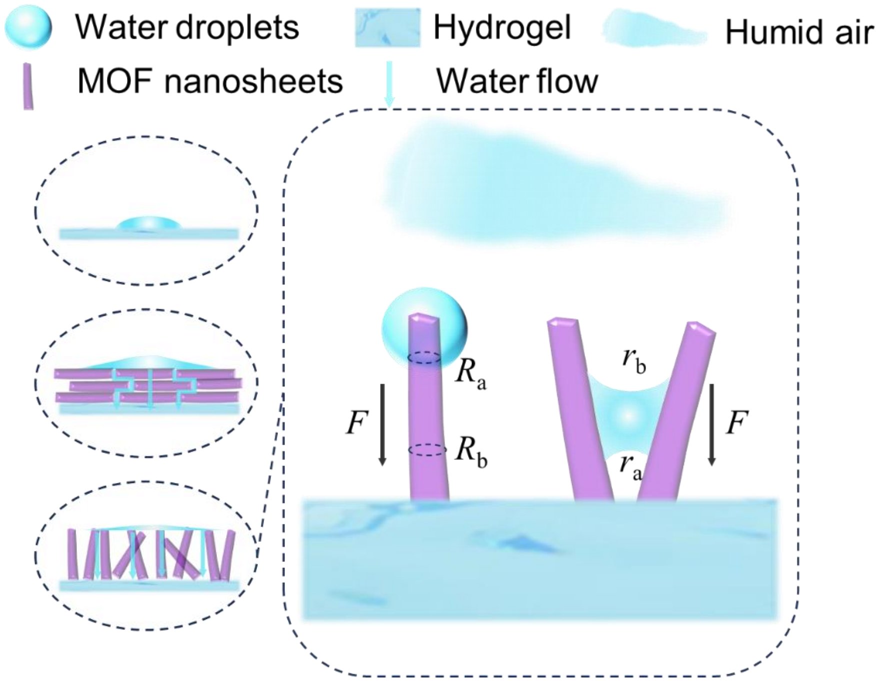

Supplementary Fig. 15. Schematic of water condensation and transportation process on MOF nanosheets and pores formed by vertical aligned MOF nanosheets. Inset: water transportation process of CT/PVA (top), HMOF-CT/PVA (middle), and MOF-CT/PVA (bottom).

Water vapor condensation and droplet transport are key processes that determine the fog collection performance of membrane harvester. As shown in Supplementary Fig. 15, the moisture was condensed on MOF nanosheets based on the capillary condensation caused by MOF porous structure. There are two critical transportation channels for the condensed water droplets to the CT/PVA substrate: through MOF nanosheets or pores formed by vertical aligned MOF nanosheets. For the first circumstance (one nanosheet model on the left), the droplets will be transported from the tip to the base of the MOF nanosheets due to the effect of the Laplace pressure difference, where R_a and R_b are the local radius of the nanosheet at two opposite sides of the droplet. For the nanosheets cluster model, r_a and r_b represent the transverse radius at the two opposite menisci. The transportation process and dynamics of various nanosheets distribution were further simulated in a simplified two-dimensional flow field (Supplementary Fig. 17 and Fig. 3b).

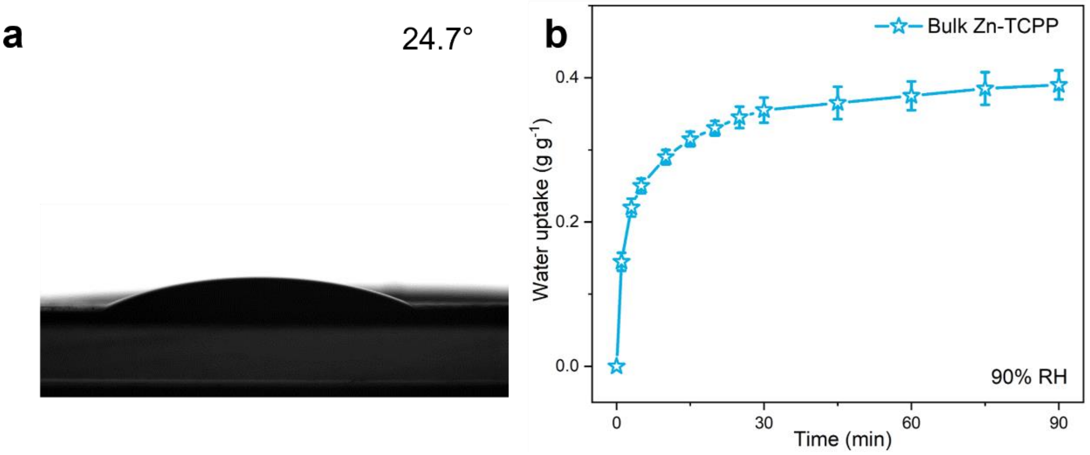

Supplementary Fig. 16. a, Water contact angle and **b,** water uptake of pure Zn-TCPP with bulk morphology. The error bars represent the standard deviation of data from three distinct samples ($n = 3$).

As shown in Supplementary Fig. 16b, we tested the water adsorption performance of pure Zn-TCPP. It is worth noting that only bulk Zn-TCPP could be fabricated without PEG as surfactants. The bulk Zn-TCPP powder was compressed into a tablet to prevent dispersion in the controlled climate chamber. Although the specific surface area of bulk Zn-TCPP is relatively lower than that of Zn-TCPP nanosheet, the water adsorption performance of pure bulk Zn-TCPP exhibited significant water uptake and kinetics (0.39 g g^{-1} water uptake within 90 mins), which is comparable to pure MOF (Supplementary Table 4).

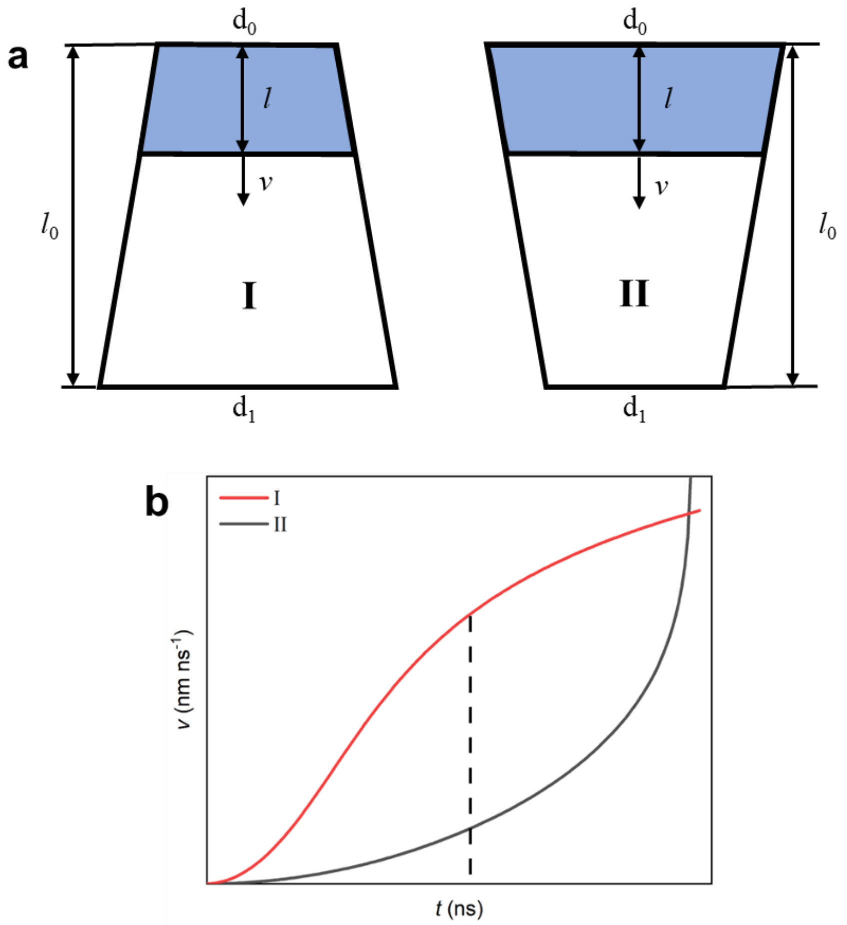

Supplementary Fig. 17. a, Dynamics simulation methods and **b**, results of water droplets transportation between MOF nanosheets. d_0 is the entry length, d_1 is the outlet length, l is the transport distance in the normal direction, l_0 is the total distance, v is the surface tension coefficient.

The simulation results provided insight into the dynamics of water droplet transportation through pore channels of MOF nanosheets. The capillary pressure difference caused by the distribution of vertically aligned nanosheets results in different dynamics of water droplets during flow from the channel top to the CT/PVA hydrogel substrate. These results explain that, when water droplets coalesce to form a continuous flow at the top of the MOF layer (Fig. 3b), a smaller initial aperture (type I, Supplementary Fig. 17a) often exhibits higher flow velocity and can quickly reach the hydrogel substrate. However, as the vertical distance of the aperture increases, the capillary pressure difference between the top and bottom causes a higher flow velocity in type II pores (Supplementary Fig. 17a).

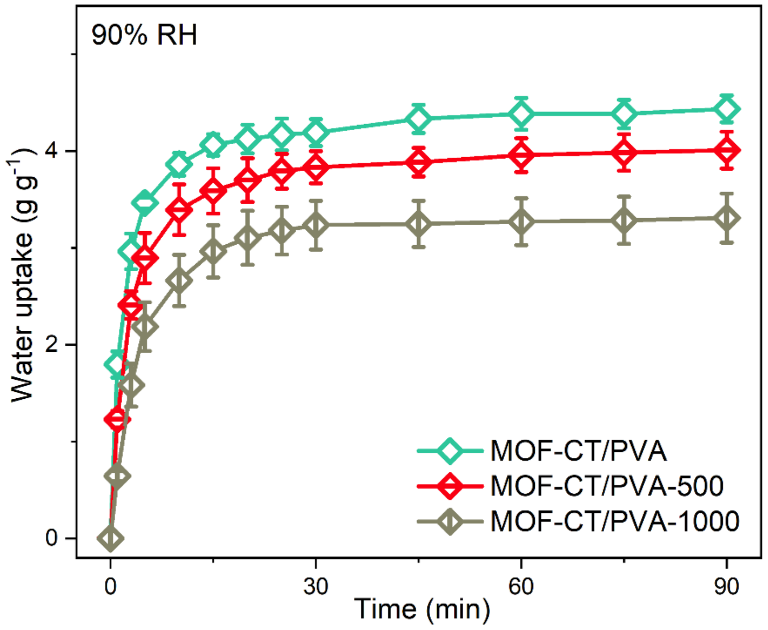

Supplementary Fig. 18. The water vapor collection performance of MOF-CT/PVA with different casting thickness of 100 μm, 500 μm, and 1000 μm. D The error bars represent the standard deviation of data from three distinct samples ($n = 3$).

We fabricated MOF-CT/PVA with various casting thickness (100 μm, 500 μm, and 1000 μm). Supplementary Fig. 18 shows that, as the thickness of the membrane substrate increased, the water uptake of MOF-CT/PVA decreased progressively. Despite this trend, MOF-CT/PVA-1000 maintained a high water uptake of 3.31 g g^{-1} at 90% RH even with a casting thickness of 1000 μm. It is worth noting that the total amount of water adsorption per membrane area significantly increased with the increase of membrane thickness, with MOF-CT/PVA-1000 achieving 226.2 g m^{-2} compared to 44.4 g m^{-2} for MOF-CT/PVA (thickness = 100 μm). These results highlight the potential for significant practical applications of the relatively thick membrane MOF-CT/PVA-1000 in water vapor harvesting.

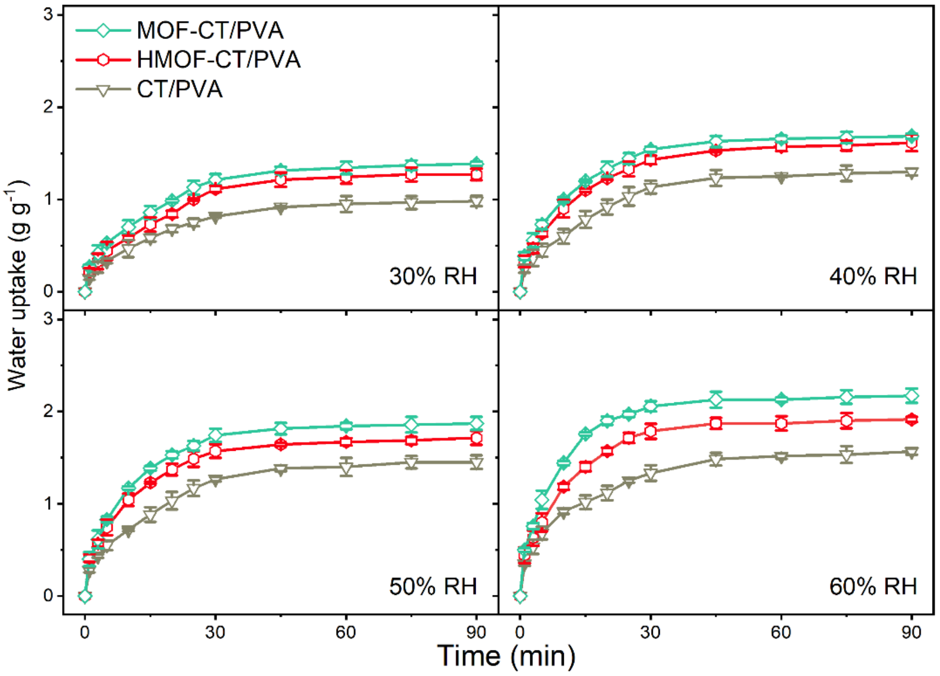

Supplementary Fig. 19. The water vapor collection performance of CT/PVA, HMOF-CT/PVA, and MOF-CT/PVA at relatively low RH (30%, 40%, 50%, and 60%). The error bars represent the standard deviation of data from three distinct samples ($n = 3$).

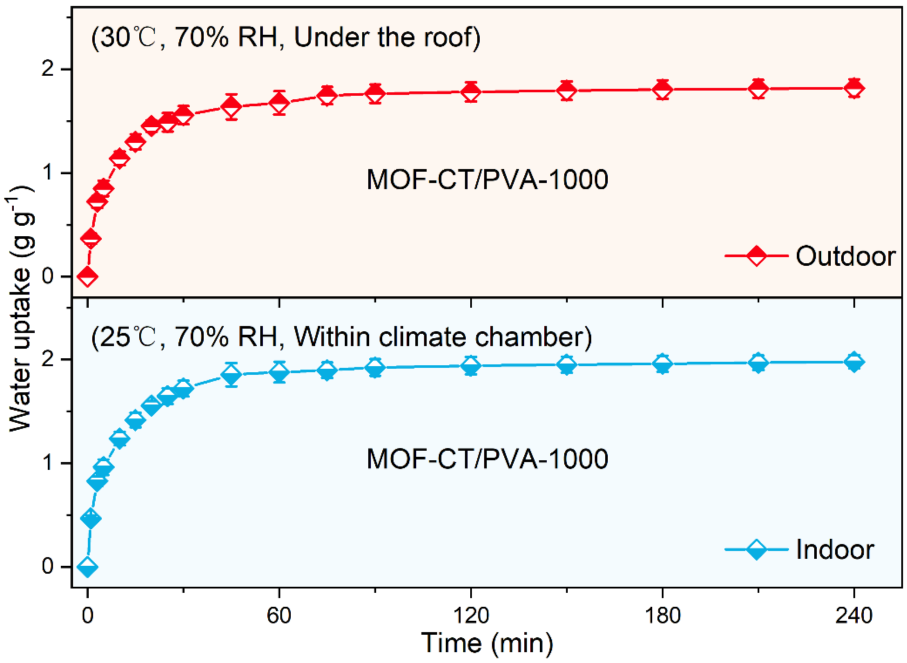

Supplementary Fig. 20. The outdoor and indoor water vapor collection performance of MOF-CT/PVA with a casting thickness of 1000 μm. The error bars represent the standard deviation of data from three distinct samples ($n = 3$).

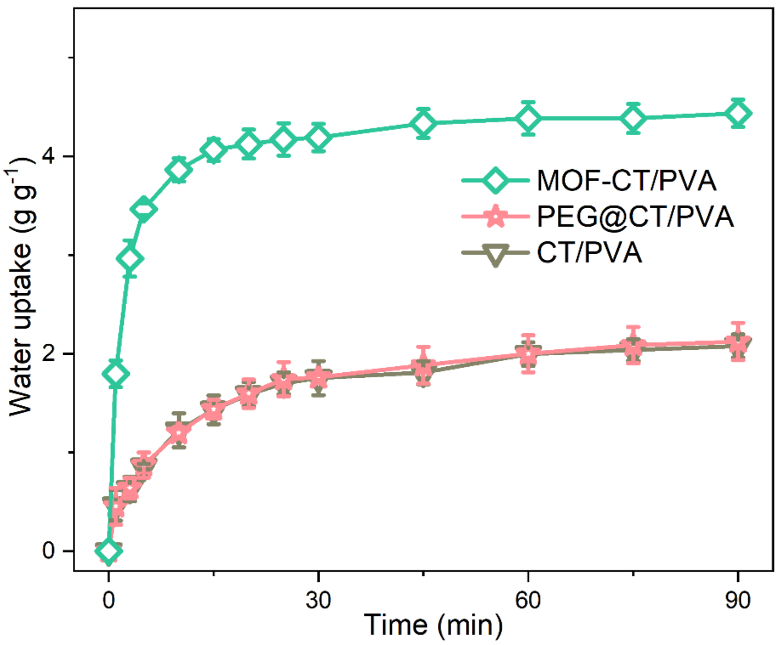

Supplementary Fig. 21. The water vapor collection performance of CT/PVA, PEG@CT/PVA, and MOF-CT/PVA at 90% RH. The error bars represent the standard deviation of data from three distinct samples ($n = 3$).

Supplementary Fig. 21 shows similar water vapor adsorption behavior for CT/PVA and PEG@CT/PVA, suggesting that PEG alone had limited influence on vapor uptake. In contrast, MOF-CT/PVA had much greater water vapor adsorption, highlighting that the directional growth of MOFs notably boosts water harvesting.

Supplementary Tables

Supplementary Table 1. Properties of molecules used for hydrogel membranes.

Molecule name	Molecule weight	Structure
Chitosan	~30000	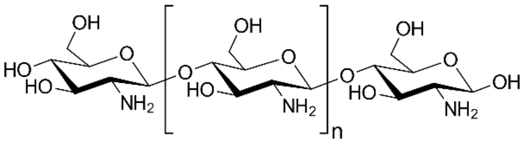
poly(vinyl alcohol)	~1700	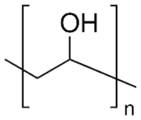
poly(ethylene glycol)	~20000	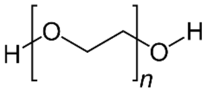

Supplementary Table 2. The XPS relative content (%) of elements.

Materials	C 1*s*	N 1*s*	O 1*s*	Zn 2*p*
Nanosheet Zn-TCPP	79.10	1.73	18.20	0.97
Bulk Zn-TCPP	74.82	5.84	14.95	4.39
MOF-CT/PVA	71.46	3.97	24.20	0.37
CT/PVA	58.77	6.58	34.65	-

Supplementary Table 3. The relative content (%) of C 1*s* functional groups.

Materials	C 1*s*			
	C-C	C-O/C-N	C=O	O=C-O
Nanosheet Zn-TCPP	49.46	24.79	12.70	13.05
Bulk Zn-TCPP	56.79	16.34	-	26.87
MOF-CT/PVA	45.93	18.16	22.58	13.33
CT/PVA	25.48	41.91	16.34	16.23

Supplementary Table 4. Performance comparison of state-of-the-art water harvesters. The performance of all listed harvesters was evaluated based on the absorbent weight and tested under 90% RH.

AWH absorbent	RH (%)	Water uptake ($g\ g^{-1}$)	Sorption rate ($g\ g^{-1}\ h^{-1}$)	Reference
MOF-801	90	0.40	0.20	5
MOF-841	90	0.45	0.23	5
UiO-66	90	0.42	0.21	5
PIZOF-2	90	0.70	0.35	5
PAMPS-CNT-LiCl	90	4.50	0.45	6
GPDDA	90	1.10	3.30	7
Cr-soc-MOF-1	90	1.95	0.98	8
SMAG	90	6.00	3.00	9
HSCC-E10	90	3.75	0.313	10
NBHA	90	2.40	0.80	11
Li-SHC	90	8.46	0.71	12
PC-MOF	90	5.40	0.72	13
CT/PVA*	90	2.00	2.00	This work
MOF-CT/PVA*	90	4.38	4.38	This work

*The adsorption equilibrium times of the reported harvesters exhibit variability. In this table, the water uptake results of 1 hour for CT/PVA and MOF-CT/PVA are provided for comparing with other harvesters.

Supplementary Table 5. The characteristics of the fabricated membranes with varying thicknesses.

	Casting thickness (μm)	Size		Weight (mg)
		Length (cm)	Width (cm)	
CT/PVA	100	4.0	3.0	141.0 ± 0.2
HMOF-CT/PVA	100	4.0	3.0	143.0 ± 0.5
MOF-CT/PVA	100	4.0	3.0	143.0 ± 0.5
MOF-CT/PVA-500	500	4.0	3.0	175.0 ± 0.7
MOF-CT/PVA-1000	1000	4.0	3.0	213.0 ± 0.8

The membrane weights are presented as mean ± standard deviation ($n = 3$).

Supplementary References

1. Zhao M*, et al.* Ultrathin 2D metal-organic framework nanosheets. *Adv Mater* **27**, 7372-7378 (2015).

2. Zhu ZH, Liu Y, Song C, Hu Y, Feng G, Tang BZ. Porphyrin-based two-dimensional layered metal-organic framework with sono-/photocatalytic activity for water decontamination. *ACS Nano* **16**, 1346-1357 (2022).

3. Zhou C*, et al.* The continuous efficient conversion and directional deposition of lithium (poly)sulfides enabled by bimetallic site regulation. *Nano Energy* **98**, (2022).

4. Zhao Y, Tong X, Kim J, Tong T, Huang CH, Chen Y. Capillary-assisted fabrication of thin-film nanocomposite membranes for improved solute-solute separation. *Environ Sci Technol* **56**, 5849-5859 (2022).

5. Kim H*, et al.* Water harvesting from air with metal-organic frameworks powered by natural sunlight. *Science* **356**, 430-434 (2017).

6. Shan H*, et al.* All-day multicyclic atmospheric water harvesting enabled by polyelectrolyte hydrogel with hybrid desorption mode. *Adv Mater* **35**, e2302038 (2023).

7. Yang K, Pan T, Pinnau I, Shi Z, Han Y. Simultaneous generation of atmospheric water and electricity using a hygroscopic aerogel with fast sorption kinetics. *Nano Energy* **78**, (2020).

8. Towsif Abtab SM*, et al.* Reticular chemistry in action: a hydrolytically stable MOF capturing twice its weight in adsorbed water. *Chem* **4**, 94-105 (2018).

9. Zhao F, Zhou X, Liu Y, Shi Y, Dai Y, Yu G. Super moisture-absorbent gels for all-weather atmospheric water harvesting. *Adv Mater* **31**, e1806446 (2019).

10. Shan H*, et al.* High-yield solar-driven atmospheric water harvesting with ultra-high salt content composites encapsulated in porous membrane. *Cell Rep Phys Sci* **2**, (2021).

11. Wang M*, et al.* Solar-powered nanostructured biopolymer hygroscopic aerogels for atmospheric water harvesting. *Nano Energy* **80**, (2021).

12. Shan H*, et al.* Exceptional water production yield enabled by batch-processed portable water harvester in semi-arid climate. *Nat Commun* **13**, 5406 (2022).

13. Yilmaz G*, et al*. Autonomous atmospheric water seeping MOF matrix. *Sci Adv* **6**, eabc8605 (2020).
