# Supplementary material for: Hydrogel-embedded vertically aligned metal-organic framework nanosheet membrane for efficient water harvesting
Source: Nat Commun. 2024 Nov 11;15:9738. doi: 10.1038/s41467-024-54215-z (PMC11555079; doi:10.1038/s41467-024-54215-z)
Supplement: Supplementary file 2 — Description of Additional Supplementary Files [file 41467_2024_54215_MOESM2_ESM.pdf]

Description of Additional Supplementary Files

File Name: Supplementary Movie 1
Description: Ultrafast water absorption process of MOF-CT/PVA
